# Urinary 6-sulfatoxymelatonin level in age-related macular degeneration patients

**Published:** 2009-08-21

**Authors:** Richard Rosen, Dan-Ning Hu, Violete Perez, Katy Tai, Guo-Pei Yu, Min Chen, Paul Tone, Steven A. McCormick, Joseph Walsh

**Affiliations:** 1 Department of Ophthalmology, New York Eye and Ear Infirmary, New York, NY; 2 Department of Pathology, New York Eye and Ear Infirmary, New York, NY; 3 Department of Biostatistics and Epidemiology Service, New York Eye and Ear Infirmary, New York, NY; 4 Departments of Ophthalmology and Otorhinolaryngology, New York Medical College, Valhalla, NY; 5 Department of Medicine, Richmond University Medical Center, Staten Island, NY

## Abstract

**Purpose:**

Melatonin is a potent antioxidant and free radical scavenger. It has been reported that serum melatonin level is relevant to certain aging diseases. The purpose of this study was to investigate melatonin levels in age-related macular degeneration (AMD) patients by measurement of 6-sulfatoxymelatonin levels (aMT6s), the major metabolite of melatonin in urine, and compare it with a group of age- and gender-matched controls.

**Methods:**

The first urine of the morning was collected from 43 AMD patients and 12 controls who did not have AMD. The level of aMT6s in specimens was measured by a commercial 6-sulfatoxymelatonin ELISA kit. The assay was performed by researchers, who were masked to the clinical information. To adjust for variation in the diluteness of urine, urinary creatinine level was measured and aMT6s levels were expressed as aMT6s/creatinine.

**Results:**

The level of urinary aMT6s/creatinine (mean±SD) in AMD (6.24±3.45 ng aMT6s/mg creatinine) was significantly lower than that of the controls (10.40±4.51, p=0.0128). After adjustment for various factors (age, smoking, cancer, and coronary heart disease) that may influence the aMT6s level, the odds-ratio of urinary aMT6s comparing AMD patients to controls was 0.65 (95% confidence interval=0.48–0.88, p=0.0036), indicating that urinary aMT6s level in AMD patients was lower than in controls even after multivariate adjustment.

**Conclusions:**

Urinary aMT6s level in AMD patients was 40% lower than in age- and gender-matched controls. This difference between AMD patients and controls is present after adjustment for the factors of age, smoking, and histories of cancer and coronary heart disease. The significance of this result and the role of melatonin in the occurrence of AMD require further investigation.

## Introduction

Melatonin, a neurohormone, is mainly produced in the pineal gland. Melatonin is involved in control of circadian rhythms (including sleep-wake cycle and other biologic rhythms), regulation of various physiologic functions, e.g., cardiovascular system, immune system, the aging process, etc [1-4]. Physiologic and pharmacological doses of melatonin are effective as an antioxidant and free radical scavenger [4-12]. Results have been published indicating that melatonin displays antioxidant capacity in several experimental conditions [4,9-22]. It has been reported that blood and urinary melatonin levels decrease with age, and a decrease of circulating melatonin has been reported in patients with age-related diseases, e.g., several types of cancer, coronary artery disease, Alzheimer disease, etc [2,4,23-41].

Age-related macular degeneration (AMD) is the leading cause of blindness in elderly persons in the western countries. Oxidative stress, the cellular damage caused by reactive oxygen species (ROS), has been implicated in the pathogenesis of AMD [32-45]. Melatonin is a strong antioxidant and can induce the expression of various antioxidant enzymes by activation of melatonin receptors. Decrease of melatonin production in aged persons may cause a reduction of antioxidant activity. Therefore, it may be useful to study the relationship between melatonin level and the occurrence of AMD.

Changes of circulatory melatonin level in AMD have not been reported previously. The purpose of this study was to examine the relationship, if any, between circulatory melatonin level and AMD by assessing nocturnal urinary excretion of 6-sulphatoxymelatonin (aMT6s, an index of peak blood melatonin concentration) in patients with AMD. A group of age- and gender-matched controls was included. In addition, the effects of factors that may influence the secretion of melatonin (age, smoking, coronary artery disease, and cancer) in these groups were evaluated carefully to exclude the possible influence caused by these factors.

## Methods

### Subjects

This study included 43 randomly selected AMD patients (21 males and 22 females) along with 12 age- and gender-matched individuals (5 males and 7 females) without AMD, who served as the controls. All individuals had normal kidney and liver functions by history. Patients were asked during examination for any history of current or previous liver or kidney diseases. Excluded were shift workers, individuals with sleep disorders were excluded; persons who had just undertaken a long distance flight, or those who had taken oral supplementation of melatonin within the last two weeks.

The criteria for diagnosis of AMD were the presence of large drusen (>125 μm), areas of macular geographic atrophy, pigmentary changes of the retinal pigment epithelium (RPE), or submacular choroidal neovascularization.

The study protocol was reviewed and approved by the New York Eye and Ear Infirmary (NYEEI) Institutional Review Board (IRB). The study adhered to the Helsinki. All participants were fully informed of the purpose and procedures of this study, and an IRB approved consent was obtained from all participants before study entry. Participants were carefully directed to collect urine on mornings following bright sunny days, which they had spent inside, and to avoid collection following sunless or overcast days for consistent environmental effect. Urine was collected at the early morning and refrigerated before delivery to the laboratory. Samples were then frozen at −70 °C until analysis.

### Measurement of 6-sulphatoxymelatonin and creatinine

The amount of 6-sulphatoxymelatonin (aMT6s) in nocturnal urine specimens was determined in duplicate by competitive enzyme-linked immunosorbent assay (ELISA). The ELISA kit was obtained from Buehlmann Laboratories AG (Schoenenbuch, Switzerland). Briefly, diluted urine samples, controls and aMT6s standards were added to wells pre-coated with antibody to rabbit immunoglobulin. Biotinylated aMT6s and rabbit anti- aMT6s antibodies were added too. aMT6s in the specimens and standards competed with biotinylated aMT6s for the binding sites of the rabbit anti-aMT6s antibody. The biotinylated aMT6s-antibody complexes formed were captured by the antibody to rabbit immunoglobulin pre-coated on the wells. The plate was shaken at a plate mixer (Labnet, Woodbridge, NJ) for 60 s at 1,000 rpm. After complete mixture on a plate mixer, the plate was incubated for 3 h at 4 °C. All wells were emptied and washed four times with wash buffer provided in the ELISA kit. Substrate solution containing hydrogen peroxide and tetramethtlbenzidine in citrate buffer was added to the well, mixed and incubated for 15 min at room temperature in the dark. The stop solution was added, and the absorbance at 450 nm of each well was read by a microplate reader (Multiskan EX, Thermo, Vantaa, Finland). The amount of aMT6s in the urine specimen was calculated following the manufacturer’s instructions. The sensitivity of the assay was 0.14% and the intra- assay and inter-assay precision were 7.1% and 11.9%, respectively. Assays of aMT6s were performed by researchers who were masked as to the identity of the patients.

Since individuals differ in the volume of urine they produce, urinary creatinine was also assayed in the present study using the kinetic Jaffe reaction [46,47]. The creatinine standard solution and picric acid were obtained from Sigma-Aldrich (St. Louis, MO). The test results were normalized using creatinine concentration and expressed as nanograms aMT6s per mg creatinine.

### Statistics

Fisher’s exact test was used to determine the statistical difference of percentages of various variables between AMD and the controls. Student *t*-test was used to examine statistical difference of means of variables between different groups. An unconditional logistic regression model was used to measure the odds ratios and 95% confidence intervals (CI) of urinary aMT6s levels between AMD and the controls. In this model, patients with AMD were coded as 1 and controls were coded as 0 for dependent variable. To control potential confounding factors, we adjusted the odds ratio by age, and histories of smoking, cancer and coronary artery disease.

## Results

Table 1 shows the demographical and clinical data of AMD patients and the controls. There was no statistically significant difference in age (p=0.1682) or gender (p=0.7507) between AMD patients and the controls. Of the participants with a history of smoking, no statistically significant difference was present in the rate of smoking history between AMD patients and the controls (p=0.1011). No participants were smoking at the time of this study. One control and four AMD patients had a history of various cancers; there was no statistically significant difference in prevalence of cancer between AMD patients and the controls (p=1.000). Two controls and 11 AMD patients had a history of coronary artery disease; the difference of prevalence of heart disease between these two groups was not statistically significant (p=0.7079).

**Table 1:** Demographical and clinical data of age-related macular degeneration patients and controls

**Factors**	**AMD**	**Control**	**p-value**
Age	75.8±9.07	73.6±6.80	0.1682
Gender (male/female)	21/22	39940	0.7507
Prior smoking	24/43 (55.8%)	3/12 (25.0%)	0.1011
Cancer history	4/43 (9.3%)	1/12 (9.1%)	1
Coronary heart disease	11/43 (25.6%)	2/12 (16.7%)	0.7079

In both AMD and the controls, some of the individuals had a history of smoking, cancer and coronary artery disease, factors that may influence the melatonin level. Statistical significance of difference in the percentage or mean of these factors between AMD and the controls was analyzed by student’s *t*-test or Fischer’s exact test, respectively. There was no statistically significant difference in these factors between AMD patients and the controls.

The amount of aMT6s in nocturnal urine was 6.24±3.45 ng aMT6/mg creatinine (mean±SD) in AMD patients, which was 60% of the level in the age- and gender-matched controls (10.4±4.51 ng/mg). The difference of aMT6 level in nocturnal urine between AMD patients and the controls was statistically significant (p=0.0128).

Table 2 shows the unadjusted and adjusted odds ratio and 95% CI of nocturnal urinary aMT6s/creatinine level as comparing AMD patients with control individuals. As shown in the table, the results of unadjusted and adjusted odds ratios were quite close, which indicates that the factors of age and histories of smoking, cancer and coronary artery disease did not significantly affect the effect of nocturnal urinary aMT6s/creatinine level between AMD patients and controls. After adjusting for age, and histories of smoking, cancer and coronary artery disease, the level of nocturnal urinary aMT6s excretion in AMD patients was significantly lower than that in the controls (adjusted odds ratio=0.65 and 95% CI=0.48–0.88, p=0.0036).

**Table 2:** Urinary 6-sulphatoxymelatonin/creatinine level in age-related macular degeneration patients and controls.

**Statistical adjustment**	**Odds ratio**	**95% CI**	**p-value**
Not adjusted for any factor	0.72	0.58–0.90	0.004
Adjusted for age only	0.73	0.58–0.91	0.006
Adjusted for smoking history only	0.7	0.53–0.89	0.005
Adjusted for histories of cancer and coronary heart disease only	0.7	0.55–0.89	0.003
Adjusted for all the above factors	0.65	0.48–0.88	0.005

The odds-ratios and 95% confidence intervals (CI) of urinary aMT6s levels between AMD and the controls were estimated by an unconditional logistic regression model. To control potential confounding factors, the odds-ratio and CI were adjusted by age, and histories of smoking, cancer and coronary artery disease using multiple logistic regression model. The difference of aMT6s level between AMD patients and the controls was still present after adjusting for all these factors, indicating that urinary aMT6s level in AMD patients is significantly lower than that of the controls.

In the AMD group, four patients had vision equal to or less than 20/400 in both eyes. Urinary aMT6s level was 5.84±2.74 ng aMT6s/mg creatinine, which was not significantly different from those who had vision better than 20/400 (6.29±5.54, p=0.778).

## Discussion

The secretion of melatonin by the pineal body is circadian, with high levels at night and very low secretion during daylight. The measurement of melatonin secretion requires drawing blood at different times during the night to measure plasma melatonin levels. This procedure is inconvenient and requires hospitalization, which is seldom feasible [48-51]. Collection of the first morning urine and measurement of melatonin level in the urine makes the collecting procedure easier. However, only less than 1% of melatonin appears unaltered in urine; most of melatonin undergoes conversion to metabolites before appearing in the urine [49]. Circulating melatonin undergoes hepatic metabolism to 6-hydroxymelatonin, which is immediately conjugated to yield 6-sulphatoxymelatonin. This product is then excreted in the urine and accounts for more than 70% of the melatonin secreted. Concentration of aMT6s in urine is 2–3 orders of magnitude higher than that of melatonin. Therefore it is very sensitive and thus no extraction is required [48,49]. It has been reported that there is a good correlation between nocturnal urinary aMT6s and plasma melatonin level during the night [48-55]. To compensate for the variation in the diluteness of urine, urinary creatinine should be used to standardize the samples for comparison. aMT6s levels in the urine can then be expressed as ratio of aMT6s/creatinine [48,52]. Estimation of nocturnal circulating melatonin levels by measuring nocturnal urine aMT6s/creatinine is a well established, reliable, and highly feasible procedure for clinical study. This procedure has been used widely for the study of melatonin secretion and its relationship with various physiologic and pathological situations [24,30,48,52,56-58].

Melatonin levels in AMD patients have not been reported. The present study compared the nocturnal urine aMT6s/creatinine levels in AMD patients to those in a group of age-matched controls to examine whether AMD was associated with a greater decrease in melatonin levels than seen in non-AMD aged individuals.

In the present study, nocturnal urine aMT6s level in AMD was significantly less than that of age-matched controls, suggesting that AMD is associated with a greater decrease of melatonin than typically seen with the normal aging process. In both AMD and the controls, some of the individuals had a history of smoking, cancer, or coronary artery disease—factors that may influence the melatonin level [1-4,25,26,29,31]. However, statistical analysis indicates that there was no significant difference in these factors between AMD patients and the controls. Furthermore, adjusted odds ratio using multiple logistic regression model also documented that the difference of aMT6s level between AMD patients and the controls was still present after adjusting for all these factors, indicating that urinary aMT6s level in AMD patients is significantly lower than that of the controls.

There are two possibilities in the causal-relationship between the lower melatonin level and AMD. First, AMD interferes in the production of melatonin, and the pathologic process in the retina is the cause of low circulating melatonin levels in AMD. Second, deficiency of melatonin plays a role in the pathogenesis and is at least one of the risk factors for AMD.

Melatonin is mainly produced in the pineal body, although, aside from ocular tissues, there are other tissues where the production of melatonin has been detected [59-63]. However, melatonin produced in the retina is principally for local purposes. Locally produced melatonin may lead to a relatively high melatonin concentration surrounding photoreceptors, thus protecting these cells either via antioxidant activity or by activation of melatonin receptors. The amount of melatonin produced in the eye is far less than that produced in pineal body [60,62,63]. It has been well documented that the blood melatonin level is derived exclusively from the pineal gland in mammals [4,59,61]. Therefore, it does not seem possible that the decrease of urinary aMT6s is due to the changes of melatonin production in the AMD eye.

Totally blind people may lose synchronization of circadian rhythms to day/night cycle [64]. However, AMD almost never leads to total blindness. In the present series, none of the AMD patients had lost light perception. In four patients with vision of both eyes at hand motion to 20/400, urinary aMT6s levels did not differ from patients with vision better than 20/400. This indicates that the decrease of urinary aMT6s in AMD is not caused by the decreased visual acuity.

There is growing evidence that cumulative oxidative stress could play an important role in the development of AMD [32-45]. Experimental animal and in vitro studies have demonstrated that various ROS can cause damage to the RPE, increase the production of vascular endothelial growth factor [65], and induce deposits beneath the RPE layer that simulate the appearance of drusen [66]. Cumulative prolonged oxidative damage at the RPE level may contribute to the development of certain anatomic changes characteristic of AMD [67,68]. Changes in blood antioxidants and lipid peroxidation products have been found in AMD patients [34,39-43]. A decline of plasma glutathione has been detected in AMD patients [69,70]. Several randomized controlled clinical trials found a beneficial effect with dietary supplementation of antioxidants on the occurrence or progression of AMD [32,36-38].

Melatonin is a free radical scavenger and antioxidant, which was first demonstrated by Ianas [71]. Since then there have been hundreds of publications that demonstrate the free radical scavenging and antioxidant actions of melatonin both in in vitro and in vivo studies [5-12]. Several melatonin oxidation products, e.g., N^1^-acetyl-N^2^-formyl −5-methoxykynuramine (AMAK) and N^1^-acetyl-5-methoxykynuramine (AMK), are also potent free radical scavengers and antioxidants [4,6]. In addition to the direct effects, conjugates of melatonin to melatonin membrane receptors (MT1/2 receptors) can induce the expression of various natural antioxidant enzymes or to increase their activity, e.g., superoxide dismutase, glutathione peroxidase, glutathione reductase and so forth [4-7,12,72]. Numerous in vitro and in vivo studies have documented the ability of both physiologic and pharmacological concentrations of melatonin to protect various cells against oxidative stress [13-22].

In the retina, melatonin receptors have been detected in the RPE, photoreceptors, retinal ganglion cells, horizontal cells and amacrine cells [59]. Melatonin may protect the RPE and photoreceptors via direct antioxidant activity and also may induce the expression of various antioxidant enzymes by activation of melatonin receptors [72-75].

Decreased production of melatonin in AMD relative to age-matched controls demonstrated in the present study indicates that deficiency of melatonin may play a role in the occurrence of AMD. The mechanism of lower melatonin level in AMD, whether it is determined by genetic or environmental factors, is unknown and requires further investigation. In addition, melatonin can also be produced by ocular cells [59,60]. Therefore, circulating melatonin level is only one of the factors determining melatonin levels in the eye.

Melatonin has been reported to protect the RPE and photoreceptors against oxidative processes [73-75]. However, the results are conflicting, and whether the protective effect of melatonin on ocular cells was due to a direct antioxidant effect or to indirect receptor-mediated effect was not distinguished. Therefore, a comprehensive study of the protective effects of melatonin on various ocular cells against different oxidative stresses and the mechanisms that are involved in these processes is required.

Yi et al. reported a clinical trial in which they tested the effects of melatonin supplementation for the treatment of AMD. It was their conclusion that melatonin protected the retina and delayed progression of macular degeneration [76]. However, this was a nonrandomized controlled study with only six months of follow-up. Furthermore, it has been reported that melatonin treatment appears to have a detrimental effect on photoreceptor cell survival in response to bright light [77]. Therefore, the time for melatonin application during the day should be considered before the organization of a clinical trial.
